# Exacerbation of Congenital Hydronephrosis as the First Presentation of COVID-19 Infection in Children

**DOI:** 10.1155/2022/9562671

**Published:** 2022-05-04

**Authors:** Masoumeh Mohkam, Mahnaz Jamee, Farshid Kompani, Mitra Khalili, Atena Seifi, Leily Mohajerzadeh

**Affiliations:** ^1^Pediatric Nephrology Research Center, Research Institute for Children's Health, Shahid Beheshti University of Medical Sciences, Tehran, Iran; ^2^Department of Pediatric Nephrology, Golestan University of Medical Sciences, Golestan, Iran; ^3^Department of Pediatric Radiology, Mofid Children's Hospital, Shahid Beheshti University of Medical Sciences, Tehran, Iran; ^4^Pediatric Surgery Research Center, Institute for Children's Health, Shahid Beheshti University of Medical Sciences, Tehran, Iran

## Abstract

**Background:**

Congenital hydronephrosis is one of the most common abnormalities of the upper urinary tract, which can be exacerbated by a variety of intrinsic or extrinsic triggers. The urinary tract system is one of the major organs complicated by COVID-19 infection. *Case Presentations*. Here, we report five patients with an established diagnosis of congenital hydronephrosis, who presented with acute abdominal pain and fever and an abrupt increase in the anteroposterior pelvic diameter (APD). Patients had a previous stable course and were under regular follow-up with serial ultrasonographic studies. They underwent surgery or supportive treatment due to the later exacerbation of hydronephrosis. Based on the clinical and imaging findings, no plausible etiologies for these exacerbation episodes, including infection, nephrolithiasis, or abdominal masses, could be postulated. The common aspect in all these patients was the evidence of a COVID-19 infection.

**Conclusions:**

Infection with COVID-19 in children with antenatal hydronephrosis may exacerbate the degree of hydronephrosis and renal APD in ultrasonography, which itself may be mediated by the increase in inflammatory mediators.

## 1. Background

Congenital hydronephrosis is one of the most common abnormalities of the upper urinary tract diagnosed by prenatal ultrasonography. Hydronephrosis, as defined by a renal pelvic diameter of more than 5 mm according to the Bristol group, is found in approximately 1–5% of all pregnancies [1]. Now, it has become clear that 70–98% of the mild isolated congenital hydronephrosis cases improve spontaneously during follow-up without any need for surgical intervention [2].

More than forty percent of congenital hydronephrosis cases are due to transient etiologies [3]; physiologic etiologies comprise 15%, ureteropelvic junction obstruction (UPJO) 11%, and vesicoureteral reflex 9% of etiologies, while other etiologies are encountered less frequently [4]. Based on the anteroposterior pelvic diameter (APD) grading system (classified as grades 1 to 4 over a scale of 5 mm), hydronephrosis grades 1 to 4 are found to resolve in 80.0%, 41.2%, 13.1%, and 2.5% of patients over a 4-year-period, respectively [5].

The most common types of postnatal hydronephrosis are the transient and physiologic ones in which a close follow-up with serial ultrasonographic studies is required if the first renal function tests are normal and no unitary tract infection (UTI) or hypertension is present.

Antibiotic prophylaxis is recommended especially in moderate to severe cases of hydronephrosis [4] and may be associated with an improvement rate of 47% in a 12-month follow-up [6].

An acute increase in the APD sometimes occurs with accompanying complications in mild to moderate cases of hydronephrosis following infections, urolithiasis, blood clots, trauma, abdominal masses, fibrosis, retroperitoneal lymphadenopathy, and malignancies.

In the present era of widespread COVID-19 infection, a heterogeneous spectrum of manifestations is plausible in the setting of COVID-19. The renal manifestations of COVID-19 in the acute phase usually range from mild proteinuria, leukocyturia, or asymptomatic hematuria to severe acute kidney injury and blood pressure changes [7, 8].

Herein, we aim to report the presentation of severe hydronephrosis in children with a previous stable mild postnatal hydronephrosis, complicated by COVID-19 infection.

## 2. Case Presentations

### 2.1. Case 1

A 6-year-old male was referred to the Emergency Department of Mofid Children's Hospital with complaints of abdominal pain and vomiting in the 2020 late spring.

The case was under follow-up due to congenital hydronephrosis in our nephrology clinic. On neonatal ultrasonography, the APD of the renal pelvis was 13 mm on the right and 12 mm on the left. The blood pressure, renal function tests, urinalysis, and voiding cystourethrography (VCUG) were normal. The APD had gradually decreased to 6 mm on the right and 5 mm on the left at 5 years of age (6 months before the last admission) in our serial follow-ups together with a normal bladder appearance and postvoiding residual volume.

On admission, the weight was 22 kg (65% percentile) and blood pressure was 100/60 mmHg. On physical examination, there was mild generalized abdominal and right costovertebral angle tenderness and there was no fever or evidence of a respiratory or another gastrointestinal problem.

The hemoglobin level was 12.8 g/dl and C-reactive protein (CRP) was in the normal range and urinalysis showed 3-4 RBCs with no WBC or protein.

Abdominal ultrasonography was performed on the first day of admission which showed the size of the kidneys to be 108 mm on the right and 80 mm on the left and severe hydronephrosis on the right with the APD of 35 mm. The APD of the left kidney and the echogenicity of both kidneys were normal. The ultrasonography of the urinary tract was repeated after 2 days and on the last imaging, the APD increased to 45 mm while clinically abdominal pain persisted without any localized tenderness during this time.

Spiral abdominopelvic computerized tomography (CT) scan showed the same findings on ultrasonography without detection of urolithiasis. TC-99m diethylenetriamine pentaacetate (DTPA) scan showed obstructive hydronephrosis in the right kidney.

The patient underwent surgery due to symptomatic obstruction and the findings during surgery were moderate obstruction and marked inflammation at the ureteropelvic junction (UPJ) without complete obstruction. He became febrile the day following the surgery, and the evaluations showed a positive CRP and also a positive polymerase chain reaction (PCR) test for the SARS-CoV-2 virus.

The chest CT scan showed the characteristic pulmonary involvement in favor of COVID-19 infection. The patient was transferred to the COVID-19 ward and was discharged after one week with a good general condition.

On the day of discharge, the ultrasonography showed mild hydronephrosis with an RPD of 8 mm and also normal laboratory tests and blood pressure.

The patient was followed quarterly for one year and showed no abnormal clinical or laboratory findings.

### 2.2. Case 2

A 13-month-old female was referred to the outpatient clinic of our hospital with complaints of mild fever and abdominal pain in February 2020. Urinalysis and urine culture were performed due to a prior history of prenatal mild hydronephrosis with a stable clinical course. Urine analysis showed pyuria, and oral cefixime was prescribed with a presumed urinary tract infection diagnosis. The patient became afebrile within two days and began to develop diarrhea and vomiting. Further evaluations showed a positive PCR test for COVID-19. The patient underwent abdominal ultrasonography which reported a severe degree of hydronephrosis.

The patient underwent pyeloplasty due to the uptake defect on the DMSA (dimercaptosuccinic acid) scan, signs of obstruction in the DTPA scan, and exacerbation of hydronephrosis after the recovery period of COVID-19 infection.

### 2.3. Case 3

A 3-year-old female was followed up due to a history of mild hydronephrosis and antireflux surgery at one year of age. She had no history of UTI and was quite stable clinically with only mild unilateral hydronephrosis on renal ultrasonography two months before referral in July 2020.

She was referred due to fever for a few days and was admitted with a presumed diagnosis of UTI due to pyuria and proteinuria in urinalysis. On repeat ultrasonography, severe hydronephrosis was detected in the ipsilateral kidney, and on the DMSA scan, a severe uptake defect of the hydronephrotic kidney was detected. In recent history, the child had exposure to a case of COVID-19 one week before admission. The patient is now well and in stable health condition.

### 2.4. Case 4

The patient was a 6-year-old female who was under follow-up due to prenatal hydronephrosis and one episode of UTI. There was no evidence of vesicoureteral reflux in her infantile evaluation.

Pyeloplasty was performed at one year of age due to the obstructive pattern on the DTPA scan. At her postsurgical follow-up, the APD in renal ultrasonography was normal and the clinical course was stable.

Three months after the last visit (in November 2020), she was referred to our outpatient clinic due to occasional complaints of abdominal pain and anorexia. In the first evaluation, urinalysis was normal and on ultrasonography, a severe degree of hydronephrosis was reported. The past medical history revealed infection with COVID-19 a week before referral.

The DTPA scan showed a nonobstructive pattern despite the severe hydronephrosis.

The patient is now under follow-up with decreasing APD in serial ultrasonography and normal clinical and laboratory data.

### 2.5. Case 5

The case was a 5-year-old male who was under follow-up due to bilateral hydronephrosis from birth. There was no history of UTI and no evidence of vesicoureteral reflux in the neonatal evaluation. Her evaluation in infancy showed a nonobstructive pattern on DTPA scan and serial follow-up revealed improving course bilaterally.

Six months before the last visit, renal function tests, urinalysis, and blood pressure were normal and the renal ultrasonography showed an improving trend of hydronephrosis.

On the last visit in May 2021, kidney ultrasonography showed a marked bilateral increase in the APD despite a stable clinical course and the history revealed infection with COVID-19 virus one month before referral. A DTPA scan was ordered and is being awaited.

## 3. Conclusions

Ureteropelvic junction obstruction (UPJO), classified as congenital anomalies of the kidney and urinary tract (CAKUT) with an obstructive pattern, constitutes the most important etiology of hydronephrosis and also one of the common causes of chronic kidney disease (CKD) in children. Severe UPJO is associated with obstruction to urine flow from the renal pelvis to the ureter and can result in ultimately complete loss of the involved kidney [9].

In childhood, UPJO is usually congenital and can be diagnosed by the prenatal kidney and urinary tract ultrasonography (usually in the 3^rd^ trimester). The acquired cases of UPJO are divided into intrinsic and extrinsic etiologies. The extrinsic causes are associated with outside pressure to the ureteropelvic junction or proximal ureters elicited by fibrosis, mass, or retroperitoneal lymphadenopathy.

The intrinsic causes of UPJO result from urolithiasis, radiation, or chronic inflammation, which cause scars in the ureteral wall or paraureteral region. According to some studies, one of the important etiologies for exacerbation of UPJO is urolithiasis, especially in the COVID-19 pandemic. In a recent study in Turkey on 149 patients with ureteral stones, it was shown that the number of emergency cases with admission during the COVID-19 epidemic increased by 3-fold in comparison with the time before the epidemic. This finding together with the simultaneous higher incidence of severe hydronephrosis in these patients can be the result of the delay in referral to the clinic as a result of personal isolation. This delayed referral can also explain some cases with a higher creatinine level [10].

The inflammatory mechanisms can be a predisposing factor for UPJO among the different abovementioned etiologies. Moreover, UPJO itself can trigger the production of vasoactive peptides and cytokines such as interleukin-5 and eotaxin-2 which recruit leukocytes and form inflammatory cell infiltrate [11].

Several studies have been conducted to evaluate the relationship between inflammatory mechanisms and UPJO over the last decades. In a study by Chiou et al. in 2005, the pathology of 24 ureteropelvic junction segments of 24 patients was evaluated. They noted an increased expression of IL-5, and eotaxin, and increased urothelial mast cell degranulation. The study showed increased levels of IL-5, *γ*-interferon, and eotaxin in the kidneys with an obstructed system, among which IL-5 and *γ*-interferon were highly (*p* < 0.05) associated with the severity of obstructive uropathy [12]. In an animal-model study by Chen et al., it was shown that the establishment of unilateral ureteral obstruction is associated with increased expression of IL-33 in vimentin-positive cells of medullary layers, cortex, and UPJ stroma. They proposed that the IL-33/ST2 axis may mediate the activation of innate immune responses and results in urothelial hyperplasia [13].

Infection with SARS-CoV-2 may result in persistent endothelial inflammation in different organs such as the lung, heart, and kidneys [14]. According to different studies, there is a marked increase in proinflammatory cytokines like ILIB, IL-6, IL-12, IFN-*γ*, IP-10, and MCP-1 in the serum of infected patients [15].

We think this marked and generalized increase in the inflammatory state may predispose patients to the exacerbation of UPJO as was observed in our patients with prior histories of COVID-19 infection.

Infection with SARS-CoV-2 in children with antenatal hydronephrosis may exacerbate the degree of hydronephrosis and renal APD in ultrasonography which itself may be mediated by the increase in inflammatory mediators.

This study has several limitations that should be considered, including lack of PCR test results, histopathologic evaluation of renal biopsies, and renal DTPA/DMSA scans for all patients. However, this is the first study that suggests COVID-19 infection as a predisposing factor for hydronephrosis exacerbation in the pediatric population. Further studies are required to confirm these clinical observations and identify the underlying pathomechanisms.

## Data Availability

Not applicable.

## Abbreviations

APD: Anteroposterior pelvic diameter
CAKUT: Congenital anomalies of the kidney and urinary tract
CKD: Chronic kidney disease
CRP: C-reactive protein
CT: Computerized tomography
DMSA: Dimercaptosuccinic acid
DTPA: Diethylenetriamine pentaacetate
IL: Interleukin
PCR: Polymerase chain reaction
UPJO: Ureteropelvic junction obstruction
UTI: Unitary tract infection
VCUG: Voiding cystourethrography.
